# Urinary Albumin-to-Creatinine Ratio Across Phenotypes of Polycystic Ovary Syndrome: A Phenotype-Based Evaluation

**DOI:** 10.3390/metabo16070448

**Published:** 2026-06-25

**Authors:** Oznur Oner, Canan Akkus, Doga Demircioglu, Ilhan Karanlık, Cevdet Duran

**Affiliations:** 1 The Department of Obstetrics and Gynecology, The Medical School of Usak University, Usak 64200, Türkiye; oneroznur@hotmail.com; 2 The Department of Internal Medicine, The Medical School of Usak University, Usak 64200, Türkiye; drilhan78@gmail.com; 3 The Department of Obstetrics and Gynecology, Usak Training and Research Hospital, Usak 64200, Türkiye; dogademircioglu@hotmail.com; 4 The Division of Endocrinology and Metabolism, The Department of Internal Medicine, The Medical School of Usak University, Usak 64200, Türkiye; drcduran@gmail.com

**Keywords:** polycystic ovary syndrome, PCOS phenotypes, albumin-to-creatinine ratio, albuminuria, microvascular involvement

## Abstract

**Highlights:**

**What are the main findings?**

- Urinary albumin excretion was not uniformly distributed across PCOS phenotypes.
- U-ACR was significantly elevated only in Phenotype B compared with controls and Phenotype D.
- Although Phenotype A had the most adverse metabolic profile, it did not show increased U-ACR.
- U-ACR was not significantly correlated with age, BMI, or HOMA-IR.

**What are the implications of the main findings?**

- Early microvascular involvement in PCOS may differ across phenotypes rather than paralleling overall metabolic severity.
- Urinary albumin assessment may therefore have phenotype-specific value for cardiovascular risk evaluation in PCOS.

**Abstract:**

**Background/Aim:** Albuminuria is a clinical marker associated with microvascular involvement and an independent predictor of cardiovascular risk. Polycystic ovary syndrome (PCOS) is associated with early metabolic and vascular abnormalities; however, whether albumin excretion differs across PCOS phenotypes remains unclear. This study aimed to evaluate the urinary albumin-to-creatinine ratio (U-ACR) across PCOS phenotypes and to examine its association with metabolic parameters. **Materials and Methods:** In this cross-sectional study, 180 women aged 18–35 years with PCOS and 51 age-matched healthy controls were included. PCOS phenotypes were classified according to the Rotterdam criteria as Phenotype A (*n* = 96), Phenotype B (*n* = 19), Phenotype C (*n* = 35), and Phenotype D (*n* = 30). Insulin resistance was assessed using the homeostasis model assessment for insulin resistance (HOMA-IR). Urinary albumin and creatinine levels were measured in morning urine samples, and U-ACR was calculated. **Results:** Age was comparable across all groups. Body mass index, waist circumference, diastolic blood pressure, and HOMA-IR were significantly higher in Phenotype A compared with controls and other phenotypes, reflecting a more adverse metabolic profile. Serum creatinine levels were similar across all groups. Despite this metabolic profile in Phenotype A, U-ACR was significantly elevated only in Phenotype B compared with controls (*p* = 0.018), and Phenotype D (*p* = 0.016). No significant correlations were observed between U-ACR and age, body mass index, or HOMA-IR. When participants were categorized according to U-ACR levels (<30, 30–299.9, and ≥300 mg/g creatinine), no significant differences in category distribution were observed between the total PCOS cohort, phenotype subgroups, and controls. **Conclusions:** Among PCOS phenotypes, U-ACR elevation was observed exclusively in Phenotype B despite similar renal function markers. This finding, in the presence of a more adverse metabolic profile in Phenotype A, suggests a dissociation between metabolic burden and early microvascular involvement across PCOS phenotypes. These findings suggest a potential phenotype-specific pattern that warrants further investigation.

## 1. Introduction

Polycystic ovary syndrome (PCOS) is one of the most prevalent endocrine-metabolic disorders affecting women of reproductive age, with a reported prevalence reaching up to 20% depending on the diagnostic criteria applied [1,2,3]. Beyond reproductive abnormalities [4], PCOS is frequently accompanied by metabolic disturbances, including insulin resistance (IR) and type 2 diabetes mellitus [5], dyslipidemia [6], and coronary artery disease [7], all of which contribute to increased long-term cardiometabolic risk.

Beyond reflecting renal involvement alone, albuminuria is increasingly recognized as a clinical parameter associated with generalized microvascular alterations and increased cardiovascular risk, and may reflect early subclinical microvascular changes [8,9,10]. Likewise, in women with PCOS, microalbuminuria has been associated with markers of microvascular involvement and increased cardiovascular risk, and has been suggested as a potential early marker of atherosclerotic risk [11,12,13,14,15,16]. Furthermore, premicroalbuminuria (defined as albumin-to-creatinine ratio > 7 mg/g in some studies) has been reported to occur more frequently in patients with PCOS compared with healthy controls and to be associated with a higher prevalence of metabolic syndrome and its individual components [17].

While previous investigations primarily focused on the presence or absence of PCOS, recent evidence emphasizes the heterogeneity of the syndrome, demonstrating that metabolic disturbances and inflammatory burden may vary substantially across different PCOS phenotypes [18]. According to the Rotterdam criteria, PCOS is classified into four phenotypes based on the presence of hyperandrogenism (HA), oligo/anovulation (OA), and polycystic ovarian morphology (PCOM): Phenotype A (HA + OA + PCOM), Phenotype B (HA + OA), Phenotype C (HA + PCOM), and Phenotype D (OA + PCOM) [18]. In general, metabolic disturbances and inflammatory burden appear to be most pronounced in the classical hyperandrogenic phenotypes and tend to decrease across the remaining phenotype categories. Accordingly, the cardiometabolic burden in PCOS may vary across phenotypes, suggesting that cardiovascular risk may not be uniformly distributed among phenotypic subgroups [19]. These observations may provide a biological basis for phenotype-specific evaluation, as differences in hyperandrogenism, metabolic burden, and inflammatory activity across PCOS phenotypes may lead to heterogeneity in early microvascular involvement.

Importantly, microvascular alterations may be influenced by mechanisms that are not fully captured by traditional metabolic markers, potentially involving phenotype-specific biological factors [20]. Thus, although metabolic disturbances tend to be more pronounced in certain PCOS phenotypes, metabolic burden alone may not fully explain early microvascular alterations. Whether urinary albumin excretion parallels metabolic severity across PCOS phenotypes or instead may be associated with a distinct phenotype-specific early vascular changes remains unclear.

Although increased urinary albumin excretion has been reported in patients with polycystic ovary syndrome, data evaluating urinary albumin excretion across different PCOS phenotypes remain limited. Therefore, the aim of this study was to evaluate the urinary albumin-to-creatinine ratio (U-ACR) across PCOS phenotypes and to explore its association with phenotype-specific differences in early vascular changes and cardiometabolic risk.

## 2. Materials and Methods

This prospective case–control study was conducted in the Departments of Internal Medicine, Endocrinology and Metabolic Diseases, and Gynecology and Obstetrics at Usak University Training and Research Hospital between 1 April 2022, and 20 July 2023. The study was performed in accordance with the principles of the Declaration of Helsinki. Ethical approval was obtained from the Ethics Committee of Medical School of Usak University (Date: 17 March 2022; Approval No: 17-17-04). Written informed consent was obtained from all participants prior to enrollment.

Women presenting with complaints such as hirsutism, acne, menstrual irregularities, or infertility and diagnosed with PCOS according to the 2003 Rotterdam criteria were included in the study based on biochemical, hormonal, and ultrasonographic evaluations.

PCOS was diagnosed when at least two of the following three Rotterdam criteria were present, (1) oligo/anovulation (OA), (2) clinical (hirsutism and/or acne) and/or biochemical signs of hyperandrogenism (HA), and (3) polycystic ovarian morphology (PCOM) on ultrasonography, after exclusion of other related clinical conditions such as Cushing’s syndrome, congenital adrenal hyperplasia, and androgen-secreting tumors. Participants were prospectively enrolled based on predefined inclusion and exclusion criteria at the time of clinical evaluation. Only women diagnosed with PCOS and without any additional systemic diseases, including hypertension, diabetes mellitus, dyslipidemia, or known renal disease, were included. Furthermore, participants were not receiving any regular medications that could affect metabolic or renal parameters. Accordingly, all individuals included in the study met eligibility criteria at enrollment, and no post hoc exclusions were applied.

**Figure 1:**
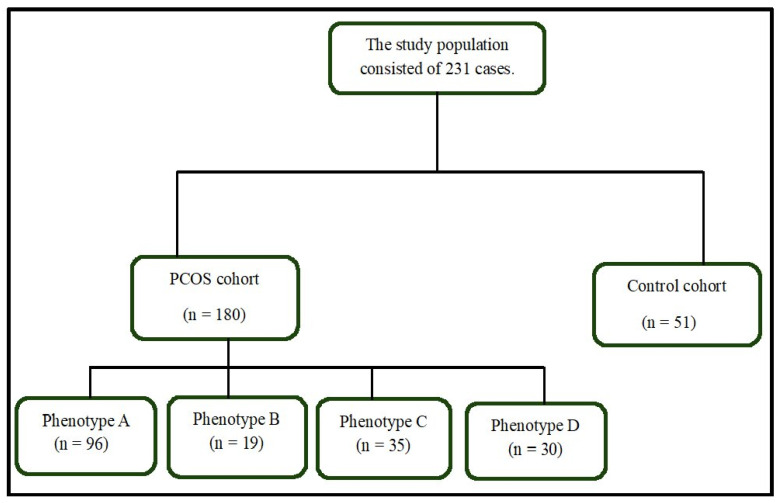
Flow diagram of the study population.

Patients were categorized into four phenotypes as follows: Phenotype A (*n* = 96), HA + OA + PCOM; Phenotype B (*n* = 19), HA + OA; Phenotype C (*n* = 35), HA + PCOM; and Phenotype D (*n* = 30), OA + PCOM (Figure 1). The control group consisted of 51 age-matched women aged 18–35 years (Figure 1) without a diagnosis of PCOS or known malignancy, liver or kidney failure, not using medications affecting insulin resistance, without active infection, hirsutism, acne, or PCOM on ultrasonography, and with regular menstrual cycles. These criteria were applied to define a clinically healthy reference group; however, no formal matching beyond age was performed.

Systolic blood pressure (SBP), diastolic blood pressure (DBP), height, weight, and waist circumference were measured in all patients and controls. No participants with diagnosed hypertension were included in the study cohort. Evaluation of hirsutism was performed by the same investigator (CD). Height (m) and weight (kg) were measured with participants wearing underwear. Waist circumference was measured at the narrowest point between the iliac crest and the lateral costal margin. Body mass index (BMI) was calculated as weight (kg) divided by height squared (m^2^).

For the measurement of androgen and gonadotropin levels, fasting blood samples were obtained during menstrual cycle days 3–5 in the morning. Serum glucose, creatinine, and insulin samples were obtained after overnight fasting. Androgen measurements were not performed in the control group. Morning urine samples for albumin and creatinine measurements were obtained during a period without menstruation. All blood samples were drawn in the morning after overnight fasting, separated by centrifugation, and stored in deep freeze at −70 °C until being analyzed.

All participants were re-evaluated for PCOM by the same examiner (OO). Ultrasonographic assessment was performed using a Mindray DC-7 device (Shenzhen Mindray Bio-Medical Electronics Co., Ltd., Shenzhen, China) with a transabdominal transducer frequency of 3–6.6 MHz and/or a transvaginal probe frequency of 3–5 MHz. PCOM was defined as the presence of ≥12 follicles measuring 2–9 mm in diameter and/or an ovarian volume > 10 mL.

Glucose levels (normal range [NR], 70–105 mg/dL) were measured using the Olympus AU 5800 analyzer (Beckman Coulter Inc., Brea, CA, USA) with the hexokinase method. Insulin levels were measured using the Immulite 2000 system (Siemens Healthcare Diagnostics, Siemens AG, Erlangen, Germany) by chemiluminescence. The intra- and inter-assay coefficients of variation for insulin were 4.6% and 5.9%, respectively.

Serum creatinine (NR, 0.6–1.1 mg/dL), urine creatinine (NR, 15–300 mg/dL), and urinary microalbumin levels were analyzed in morning urine samples. Serum and urine creatinine levels were measured using the alkaline picrate method, whereas urinary microalbumin levels were measured by the immunoturbidimetric method using the Abbott Alinity C series analyzer (Abbott Laboratories, Abbott Park, IL, USA).

IR was assessed using the homeostasis model assessment for insulin resistance (HOMA-IR), calculated using the formula: fasting plasma glucose (mmol/L) × fasting insulin (µIU/mL)/22.5.

To assess sample size adequacy, a literature-based sample size estimation was performed using G*Power software (version 3.1.9.7). Because direct phenotype-based data on urinary albumin excretion in PCOS are limited, the calculation was based on the closest recent case–control evidence. Using the difference in microalbuminuria frequency reported by Gungor et al. in women with PCOS and controls (24% vs. 4%), with a two-sided alpha of 0.05 and 80% power, the minimum required sample size was estimated to be 41 participants per group [21]. Accordingly, the final study population exceeded this requirement.

### Statistical Analysis

All statistical analyses were performed using SPSS version 22.0 (IBM Corp., Armonk, NY, USA). The normality of continuous variables was assessed using the Shapiro–Wilk test. The statistical analysis plan was defined a priori and conducted in consultation with a biostatistician.

For group comparisons, the independent samples *t*-test was used for normally distributed continuous variables, whereas the Mann–Whitney U test was applied to non-normally distributed variables. For comparisons involving more than two groups, the Kruskal–Wallis test was used, followed by pairwise comparisons when appropriate. Categorical variables were compared using Fisher’s exact test for two-group comparisons and the Pearson chi-square test for comparisons involving more than two groups. Due to the limited and uneven subgroup sizes, phenotype-stratified multivariable analyses were not performed to avoid overfitting and unstable estimates. To assess whether PCOS phenotype was independently associated with urinary albumin excretion after adjustment for potential confounders, a two-block hierarchical linear regression analysis was performed using log-transformed U-ACR values across the entire study population (*n* = 231). Given the right-skewed distribution of raw U-ACR values, natural logarithmic transformation was applied prior to analysis, as confirmed by the Shapiro–Wilk test. In Block 1, age, BMI, and HOMA-IR were entered simultaneously as covariates. In Block 2, dummy-coded PCOS phenotype variables (Phenotypes A–D) were included, with the control group defined as the reference category. The incremental explanatory value of phenotype classification beyond metabolic covariates was assessed using R^2^ change statistics. Multicollinearity was evaluated using variance inflation factors (VIF), with values < 5.0 considered acceptable. The results of the hierarchical regression analysis are presented in Supplementary Table S1.

Normally distributed continuous variables are presented as mean ± standard deviation (SD), while non-normally distributed variables are expressed as median (minimum–maximum).

Statistical significance was evaluated at a 95% confidence interval (CI), and a *p*-value ≤ 0.05 was considered statistically significant. No formal adjustment for multiple comparisons was applied; therefore, the results of subgroup analyses should be interpreted with caution.

## 3. Results

The demographic and clinical characteristics of the study population are summarized in Table 1. Age was comparable across all PCOS phenotypes and the control group.

BMI was significantly higher in the overall PCOS cohort and in Phenotypes A, B, and D compared with controls (all *p* ≤ 0.028). In addition, BMI was higher in Phenotype A than in Phenotypes C and D (*p* < 0.001 and *p* = 0.031, respectively). Waist circumference showed a similar pattern, being significantly greater in the overall PCOS group and particularly in Phenotypes A and B compared with controls (all *p* ≤ 0.041). Moreover, waist circumference was higher in Phenotype A than in Phenotypes C and D (*p* < 0.001 and *p* = 0.005, respectively). SBP values were comparable across groups, whereas DBP was significantly higher in Phenotype A compared with controls and with Phenotypes C and D (*p* ≤ 0.048).

HOMA-IR levels were significantly higher in all PCOS phenotypes compared with controls (*p* ≤ 0.024). Among the subgroups, Phenotype A exhibited higher HOMA-IR levels than Phenotypes C and D (both *p* = 0.008).

Renal and urinary parameters are summarized in Table 2. Serum creatinine levels were comparable across all groups. However, eGFR values were modestly but significantly lower in Phenotype A compared with controls and Phenotype C (*p* = 0.034 and *p* = 0.030, respectively). Urinary creatinine levels were similar in all groups.

Urinary albumin levels were significantly higher in Phenotypes A and B compared with both controls and Phenotype C (all *p* ≤ 0.022). In addition, urinary albumin levels were modestly higher in the overall PCOS cohort than in controls (*p* = 0.045).

U-ACR was significantly elevated only in Phenotype B compared with both controls and Phenotype D (*p* = 0.018 and *p* = 0.016, respectively) (Table 2).

When participants were categorized according to U-ACR levels (<30, 30–299.9, and ≥300 mg/g creatinine), no significant differences were observed in the distribution of albuminuria between the overall PCOS cohort and the control group or among the individual phenotypic subgroups (Table 3).

Correlation analysis demonstrated significant positive correlations between BMI and age (r = 0.235, *p* < 0.001) and between BMI and HOMA-IR (r = 0.537, *p* < 0.001). In contrast, no significant correlations were observed between U-ACR and age, BMI, or HOMA-IR (Table 4).

A hierarchical linear regression analysis was performed to evaluate the independent contribution of PCOS phenotype. Age (β = −0.091, *p* = 0.184), BMI (β = −0.024, *p* = 0.768), and HOMA-IR (β = 0.068, *p* = 0.391) were not significant predictors of log-transformed U-ACR when entered in Block 1. The model explained 1.3% of the variance and was not statistically significant (R^2^ = 0.013, *p* = 0.407).

When PCOS phenotype variables were added in Block 2, Phenotype B was the only variable significantly associated with log-transformed U-ACR (β = 0.164, *p* = 0.029; 95% CI: 0.066–1.240), whereas Phenotypes A, C, and D were not significantly associated with U-ACR after adjustment (all *p* > 0.20).

The inclusion of phenotype variables increased the explained variance to 4.3% (ΔR^2^ = 0.031), although this increase did not reach statistical significance (*p* = 0.134).

## 4. Discussion

Growing evidence suggests that women with PCOS exhibit early microvascular alterations that may precede overt cardiometabolic disease. The present study evaluated urinary albumin excretion across different PCOS phenotypes and showed that U-ACR levels were not uniformly distributed among phenotypic subgroups. Although overall U-ACR levels were comparable between the total PCOS cohort and controls, phenotype-specific analyses indicated that U-ACR was significantly higher in Phenotype B compared with both the control group and Phenotype D. Serum creatinine levels were similar across all groups, suggesting preserved overall renal function. Nevertheless, eGFR values were modestly but significantly lower in Phenotype A compared with controls and Phenotype C, indicating subtle differences in renal filtration parameters despite comparable creatinine levels. In line with this observation, previous studies have reported that although eGFR levels may not differ significantly between women with PCOS and healthy controls, renal filtration parameters may still show associations with metabolic factors such as BMI, insulin levels, and HOMA-IR, suggesting that early renal alterations in PCOS may be associated with metabolic disturbances [22].

From a clinical perspective, these observations highlight the importance of considering phenotypic heterogeneity when evaluating early microvascular risk markers in women with PCOS. Although U-ACR levels were not elevated across all phenotypes, the selective increase observed in Phenotype B suggests that urinary albumin assessment may have phenotype-specific value in identifying subgroups that may have increased microvascular involvement. In contrast, the absence of U-ACR elevation in the remaining phenotypes suggests that albuminuria is not uniformly distributed across the PCOS spectrum and may reflect distinct pathophysiological mechanisms within individual phenotypic profiles. However, given the relatively small sample size of Phenotype B, the absence of adjustment for multiple comparisons, and the lack of significant differences in category-based analyses, this finding should be interpreted with caution.

Correlation analysis demonstrated significant positive associations between BMI and age, as well as between BMI and HOMA-IR. In contrast, no significant correlations were observed between U-ACR and age, BMI, or HOMA-IR, suggesting that urinary albumin excretion in this cohort was not directly related to these metabolic parameters.

To further explore this observation, a hierarchical linear regression analysis was performed across the entire study population. While age, BMI, and HOMA-IR were not significant predictors of U-ACR, Phenotype B was the only variable significantly associated with U-ACR after adjustment. These findings suggest that urinary albumin excretion in this cohort may not be primarily driven by conventional metabolic factors, but rather be associated with phenotype-specific differences. However, these findings should be interpreted with caution, as no formal adjustment for multiple comparisons was performed, and category-based analyses did not demonstrate significant differences across groups, supporting the exploratory nature of this observation.

This pattern is consistent with the possibility that urinary albumin excretion may reflect a dimension of early vascular changes that is not fully captured by conventional metabolic indices. In the context of PCOS, where cardiometabolic risk is influenced by complex interactions between metabolic, hormonal, and inflammatory pathways, albuminuria may therefore provide complementary information beyond traditional metabolic markers [23].

When participants were classified according to U-ACR categories, no significant differences were observed in the distribution of albuminuria levels between the overall PCOS cohort and the control group. Likewise, comparisons between controls and individual PCOS phenotypes, as well as among the phenotypic subgroups themselves, did not reveal any significant differences in U-ACR category distribution. In a cross-sectional study of Bangladeshi women with PCOS, Kamrul-Hasan et al. reported that albuminuria (defined as U-ACR ≥ 30 mg/g) was present in approximately one-fifth of patients and was associated with certain metabolic features, suggesting a potential association between urinary albumin excretion and early cardiometabolic risk in PCOS [24].

Ziaee et al., in a study including 78 women with PCOS (mean age 27.2 ± 2.5 years) and 63 age-matched control women (mean age 26.9 ± 2.4 years), defined the threshold for premicroalbuminuria as U-ACR > 7 mg/g [17]. Using this cutoff, the authors reported that the prevalence of premicroalbuminuria was significantly higher in women with PCOS compared with controls, suggesting a higher prevalence of increased urinary albumin excretion in this population. When the PCOS cohort was further stratified into subgroups with premicroalbuminuria (U-ACR > 7 mg/g) and without premicroalbuminuria (U-ACR ≤ 7 mg/g), the subgroup with U-ACR > 7 mg/g had a higher proportion of patients with serum glucose levels ≥ 100 mg/dL, serum insulin levels > 10 μIU/mL, serum triglyceride levels > 150 mg/dL, blood pressure ≥ 130/85 mmHg, and waist circumference > 88 cm, as well as a higher prevalence of metabolic syndrome according to the NCEP-ATP III criteria. In our study, when U-ACR levels were categorized into three groups as <30 mg/g creatinine, 30–299.9 mg/g creatinine, and ≥300 mg/g creatinine, no significant differences were observed in patient distribution either among the subgroups and controls or between the overall PCOS cohort and controls.

Until recently, the presence or absence of PCOS was considered the principal determinant; however, emerging evidence suggests that metabolic alterations and inflammatory burden vary across the distinct PCOS phenotypes defined in recent years, with the severity and frequency of accompanying metabolic disturbances and inflammation decreasing as the phenotype number progresses [18]. Phenotypes characterized by the coexistence of HA and OA, such as Phenotype A and Phenotype B, are considered the most metabolically active subgroups. These findings are consistent with the possibility that microvascular alterations in PCOS may not parallel the overall metabolic severity and may instead reflect phenotype-specific biological vulnerability. Interestingly, despite the more adverse metabolic profile observed in Phenotype A, elevated U-ACR levels were not detected in this subgroup. The selective elevation of U-ACR observed in Phenotype B, despite the more adverse metabolic profile observed in Phenotype A, raises the possibility that distinct pathophysiological mechanisms may underlie renal microvascular involvement across PCOS phenotypes. From a clinical perspective, these observations highlight the importance of considering phenotypic heterogeneity when evaluating early microvascular risk markers in women with PCOS [25]. Recent studies have begun to explore renal microvascular injury and urinary biomarkers in women with PCOS. In a recent investigation, Gungor et al. reported that markers reflecting glomerular podocyte injury were more frequently detected in women with PCOS exhibiting HA and metabolic disturbances, suggesting a possible association between androgen-related metabolic activity and early renal microvascular involvement. Although these findings do not directly establish phenotype-specific differences in albuminuria, they support the concept that hyperandrogenic or metabolically active PCOS subgroups may exhibit a greater propensity for subtle renal and microvascular alterations. In this context, the selective elevation of U-ACR observed in Phenotype B in our cohort may be associated with differences in renal microvascular susceptibility across PCOS phenotypes [21]. This finding suggests that the coexistence of hyperandrogenism and oligo/anovulation in Phenotype B may be associated with phenotype-specific susceptibility to early microvascular alterations, even in the absence of the most pronounced overall metabolic burden. However, given the relatively small sample size of Phenotype B, the absence of adjustment for multiple comparisons, and the lack of significant differences in category-based analyses, this observation should be interpreted with caution.

Although phenotype-stratified multivariable modeling was not feasible due to limited and uneven subgroup sizes, an overall multivariable hierarchical regression analysis was performed across the entire cohort. Nevertheless, the observation that Phenotype A exhibited the most adverse metabolic profile, whereas U-ACR elevation was observed only in Phenotype B, together with the absence of significant correlations between U-ACR and BMI or HOMA-IR, suggests that urinary albumin excretion in this cohort may not simply parallel overall metabolic severity. However, these findings should be interpreted with caution given the relatively small sample size of Phenotype B and the absence of formal adjustment for multiple comparisons.

This apparent dissociation between metabolic burden and urinary albumin excretion may be related to phenotype-specific differences in microvascular characteristics and inflammatory activity that are not fully captured by conventional metabolic markers such as BMI or HOMA-IR. Early microvascular changes may occur through mechanisms that are partially independent of overt metabolic impairment, reflecting differences in tissue-level susceptibility across phenotypes. The lack of correlation between U-ACR and metabolic parameters in the present study is further consistent with the possibility that urinary albumin excretion may reflect a potentially distinct dimension of cardiometabolic risk rather than a direct surrogate of overall metabolic severity. Additionally, unmeasured factors such as inflammatory status and microvascular function may also contribute to this dissociation. The relatively low proportion of variance explained by the regression model further suggests that urinary albumin excretion is likely influenced by additional factors that were not assessed in the present study. Future investigations incorporating a broader range of metabolic, hormonal, inflammatory, vascular, and lifestyle-related variables may help to better characterize the determinants of U-ACR variability in women with PCOS.

In line with this observation, although the proportion of patients with U-ACR levels between 30–299.9 mg/g creatinine appeared to be higher in Phenotype B than in the other phenotypes and controls, this difference in U-ACR category distribution did not reach statistical significance according to the Pearson chi-square test. Direct phenotype-based data on urinary albumin excretion in PCOS remain limited. Moriconi et al. [26] demonstrated that in individuals with obesity, the U-ACR may underestimate the true extent of albuminuria due to increased urinary creatinine excretion. Because patients with Phenotype A in our cohort exhibited higher levels of adiposity, it is possible that albuminuria may have been partially underestimated in this subgroup when assessed using spot U-ACR measurements. Therefore, in studies involving PCOS or other obese populations, particularly phenotypes characterized by the coexistence of HA and OA (Phenotypes A and B), the assessment of albuminuria using 24-h urinary albumin excretion rather than relying solely on U-ACR may provide a more comprehensive evaluation of microvascular involvement. Additionally, the relatively small number of patients in certain subgroups in our study may have contributed to these findings. This issue may be particularly relevant in cohorts with higher levels of adiposity. Accordingly, reliance exclusively on spot U-ACR measurements may lead to an underrecognition of subtle albumin excretion abnormalities in metabolically active phenotypes of PCOS. Notably, urinary albumin concentrations were significantly higher in both Phenotype A and Phenotype B compared with controls, whereas a significant increase in U-ACR was observed only in Phenotype B. This discrepancy may be relevant when interpreting albuminuria in phenotypes characterized by greater adiposity. Given that U-ACR incorporates urinary creatinine in the denominator, higher creatinine excretion may partially attenuate the apparent degree of albuminuria in individuals with increased adiposity. This may partly explain the discrepancy between the absolute urinary albumin concentrations and the U-ACR findings observed in the present study. In such populations, complementary approaches such as 24-h urinary albumin excretion may provide a more comprehensive assessment of renal microvascular involvement and cardiovascular risk. This methodological limitation should be considered when interpreting phenotype-specific differences in U-ACR.

In line with our findings demonstrating significantly higher insulin and HOMA-IR levels in the overall PCOS cohort, as well as in Phenotypes A and B compared with controls, Borzan et al. [27] reported that IR is more pronounced in the hyperandrogenic “classical” PCOS phenotypes defined according to the Rotterdam criteria, particularly Phenotypes A and B. In their study, logistic regression analyses demonstrated a significantly increased risk of IR in these phenotypes compared with healthy controls, suggesting an important role of hyperandrogenism in shaping the metabolic phenotype of PCOS. These findings are concordant with our results and are consistent with the observation that PCOS patients with hyperandrogenism, especially those with Phenotypes A and B, may represent a subgroup with a greater metabolic burden and an increased risk of IR.

Our observation that IR, as reflected by elevated HOMA-IR levels, is significantly higher in the overall PCOS population compared with controls is further supported by a recent systematic review by Marchesan et al. [28]. In this review, the majority of PCOS-control comparative studies (13 out of 17) consistently demonstrated higher HOMA-IR values in women with PCOS, irrespective of ethnicity or study design, underscoring IR as a core metabolic feature of the syndrome. Taken together, these findings are consistent with our findings and reflect that increased IR is a reproducible and consistent feature of PCOS, particularly evident in classical and hyperandrogenic phenotypes.

Several limitations of the present study should be acknowledged. First, the cross-sectional design of the study precludes causal inferences regarding the observed associations between PCOS phenotypes and urinary albumin excretion. In addition, although participants were selected based on strict inclusion and exclusion criteria, the possibility of selection bias cannot be entirely excluded.

Second, the relatively small and uneven sample sizes across PCOS phenotypes, particularly the limited number of patients in Phenotype B, may have reduced the statistical power and reliability of between-phenotype comparisons and should be considered when interpreting phenotype-specific findings, potentially limiting the generalizability of the results. Furthermore, although adjustment for interrelated metabolic parameters such as BMI and HOMA-IR would be of interest, the present study was not designed or sufficiently powered for reliable phenotype-stratified multivariable modeling, particularly given the small and uneven subgroup sizes, including Phenotype B (*n* = 19). Under these conditions, such models would be prone to coefficient instability and overfitting and were therefore not performed to avoid potentially misleading inferences. In addition, no formal adjustment for multiple comparisons was performed, which may increase the risk of type I error and should be considered when interpreting subgroup findings. Accordingly, larger prospective studies with adequate phenotype-specific sample sizes are warranted to determine whether the observed U-ACR differences remain independent of adiposity and insulin resistance.

Third, androgen measurements were not performed in the control group, precluding direct biochemical comparisons of hyperandrogenism between PCOS phenotypes and controls. Moreover, the control group was matched only for age, and no formal matching was performed for BMI or androgen levels, which may have introduced residual confounding.

Fourth, albuminuria was assessed using a spot U-ACR rather than 24-h urinary albumin excretion, which may have led to an underestimation of albuminuria, especially in obese and metabolically active PCOS phenotypes. Participants were not given specific instructions regarding strenuous exercise or dietary intake prior to urine collection. Although urine samples were collected during a non-menstrual period and individuals with active infection were excluded, the potential influence of transient physiological factors on urinary albumin excretion cannot be completely excluded. Furthermore, thyroid function was not systematically assessed in the study population; because thyroid dysfunction may influence metabolic and renal-related parameters, this should be considered a further limitation when interpreting the findings. All participants had preserved renal function at inclusion based on serum creatinine and eGFR criteria; therefore, the relatively wide ranges observed in U-ACR may reflect the biological variability inherent to spot urine measurements rather than underlying renal pathology, and should be interpreted within the context of the cross-sectional design of the study. UA, UC, and U-ACR values were determined only after laboratory analysis; therefore, the observed variability was not known at the time of participant inclusion. In addition, all eligible participants meeting the predefined inclusion criteria were retained in the analysis. Subclinical or undiagnosed conditions cannot be entirely excluded in this cross-sectional design.

Despite these limitations, this study has several strengths. The study design, together with the inclusion of a well-characterized cohort, supports the internal validity of the findings. To our knowledge, data evaluating albuminuria across PCOS phenotypes remain limited, and the present study provides preliminary insights into phenotype-specific differences in microvascular involvement. In addition, the standardized clinical, biochemical, and ultrasonographic assessments performed by the same investigators, together with strict inclusion and exclusion criteria, strengthen the robustness and reproducibility of the results.

Elevated U-ACR levels in non-diabetic individuals and in patients with PCOS have been associated with increased cardiovascular risk and microvascular involvement. Furthermore, the presence of microalbuminuria is recognized as a marker that may be used for the early detection of atherosclerotic disease development [8,11,12,13,14,15,16]. Taken together, these findings suggest that PCOS phenotypes characterized by the coexistence of HA and OA, particularly Phenotype B, may be associated with an increased cardiometabolic risk profile. This observation highlights the potential importance of careful cardiometabolic evaluation in this subgroup and may have implications for future risk stratification strategies. In this context, recent international guidelines emphasize the importance of comprehensive cardiometabolic risk assessment in women with PCOS, highlighting that cardiovascular risk may extend beyond reproductive manifestations of the syndrome. Our phenotype-specific findings therefore are consistent with the possibility that risk evaluation in PCOS may benefit from considering phenotypic heterogeneity rather than relying solely on the presence of the diagnosis itself [23,25].

Although hyperandrogenism is a defining feature of both Phenotype A and Phenotype B, the selective elevation of urinary U-ACR in Phenotype B suggests that absolute androgen concentration alone may not fully account for early vascular changes in PCOS. However, because androgen measurements were not available in the control group, the present study cannot directly evaluate the independent contribution of hyperandrogenism to the observed differences in urinary albumin excretion. Therefore, any interpretation regarding a potential role of hyperandrogenism in microvascular alterations should be considered cautious and indirect. This observation may reflect the possibility that vascular and microvascular alterations are influenced by factors beyond total androgen levels, including differences in androgen bioavailability, receptor sensitivity, downstream signaling pathways, or phenotype-specific inflammatory and vascular responsiveness. This may help explain why Phenotype B demonstrated higher U-ACR levels despite a less adverse metabolic profile than Phenotype A. The coexistence of hyperandrogenism and oligo/anovulation in Phenotype B may represent a distinct pathophysiological profile that is more closely linked to early microvascular dysfunction than conventional metabolic markers alone. Moreover, the coexistence of more pronounced metabolic abnormalities in Phenotype A without a parallel increase in U-ACR is consistent with the possibility that renal microvascular involvement in PCOS may not be linearly related to overall metabolic burden. It is also possible that differences in microvascular responses across phenotypes may contribute to this dissociation. However, the relatively small sample size of Phenotype B (*n* = 19) represents an important limitation, as the limited number of participants may have influenced statistical stability and effect estimation. Therefore, these findings should be interpreted cautiously and considered hypothesis-generating. Future studies should combine phenotype-based urinary albumin assessment with direct markers of vascular or renal injury and longitudinal cardiovascular follow-up to determine whether the observed signal in Phenotype B may reflect a transient biochemical variation or a reproducible marker of early microvascular risk. From a biomarker perspective, these findings highlight the challenges of identifying universally applicable markers for cardiometabolic risk in PCOS. Recent evidence suggests that although numerous metabolic and inflammatory markers have been investigated, only a limited number demonstrate consistent clinical utility across different populations. In this context, our findings do not establish U-ACR as a universal marker across all PCOS phenotypes, but they suggest that urinary albumin-based assessment may have selective value in particular phenotypic contexts. Such phenotype-oriented biomarker approaches may improve early risk detection and support more individualized cardiometabolic monitoring strategies in women with PCOS [29]. Collectively, our results are consistent with the growing recognition that PCOS represents a heterogeneous cardiometabolic condition in which vascular risk may differ substantially across phenotypic subgroups. In this context, recent evidence further supports the concept that microvascular dysfunction in PCOS may be reflected by a range of biomarkers rather than a single uniform indicator. Previous studies have demonstrated that markers of renal microvascular injury, particularly those associated with hyperandrogenic and metabolically active PCOS subgroups, may be associated with increased susceptibility to early microvascular alterations [21]. In addition, contemporary evidence suggests that the clinical utility of biomarkers in PCOS may be heterogeneous and context-dependent, with no single marker consistently capturing the full spectrum of cardiometabolic risk across all phenotypes [29]. More recent studies have likewise shown that microvascular impairment in PCOS may be detectable even when conventional cardiovascular risk estimates do not differ substantially, and that circulating markers of vascular activation and fibrinolytic imbalance may remain altered independent of obesity-matched comparisons or simple metabolic correlations [30,31]. Within this broader framework, the phenotype-specific pattern observed in our study supports the notion that urinary albumin excretion may have selective relevance in certain PCOS subgroups rather than representing a universal marker across the entire syndrome. Future longitudinal and mechanistic studies are warranted to clarify whether the phenotype-specific elevation in U-ACR observed in this study represents a transient biochemical finding or a clinically meaningful predictor of subsequent cardiovascular events and microvascular complications.

## 5. Conclusions

This study provides a phenotype-based evaluation of urinary albumin excretion in women with PCOS and suggests that albuminuria is not uniformly distributed across PCOS phenotypes. While overall U-ACR levels were comparable between the total PCOS cohort and controls, a selective elevation was observed in Phenotype B, although this finding should be interpreted with caution given the limited sample size and exploratory nature of the findings.

These findings suggest that urinary albumin excretion may not simply parallel overall metabolic burden but may be associated with phenotype-specific differences in microvascular involvement. This dissociation between metabolic profile and albuminuria is consistent with the heterogeneity of PCOS and suggests that conventional metabolic markers alone may not fully capture early microvascular alterations.

Future longitudinal studies incorporating more comprehensive assessments of urinary albumin excretion, including 24-h urinary albumin measurements, may help to better characterize the onset and progression of renal microvascular alterations across different PCOS phenotypes.

In this context, phenotype-oriented evaluation may have implications for cardiometabolic risk assessment in women with PCOS. Further prospective studies with larger, phenotype-stratified cohorts are needed to determine whether these findings reflect early microvascular alterations and to clarify their potential clinical significance.

## Figures and Tables

**Table 1:** Demographic features of the study population.

	Control Group (*n* = 51)	PCOS Phenotype A (*n* = 96)	PCOS Phenotype B (*n* = 19)	PCOS Phenotype C (*n* = 35)	PCOS Phenotype D (*n* = 30)	PCOS (*n* = 180)	Significant Pairwise Comparisons
Age (year)	23 (22–33)	25 (18–35)	22 (18–34)	25 (18–35)	24 (20–35)	24 (18–35)	All comparisons NS
BMI (kg/m^2^)	21.48 (17.78–27.34)	27.19 (17.15–49.77)	24.83 (15.78–39.84)	22.14 (15.59–34.08)	24.88 (17.19–50.22)	25.49 (15.59–50.22)	A > Ctrl ***, B > Ctrl *, D > Ctrl **, A > C ***, A > D *, PCOS > Ctrl ***
WC (cm)	75 (59–100)	90 (61–129)	82 (60–120)	77 (58–105)	78 (60–116)	84 (58–129)	A > Ctrl ***, B > Ctrl *; A > C ***, A > D **; PCOS > Ctrl ***
SBP (mmHg)	110 (90–130)	110 (70–150)	110 (102–160)	110 (94–140)	110 (90–140)	110 (70–160)	B vs. D *
DBP (mmHg)	70 (50–90)	70 (50–93)	70 (60–90)	70 (50–90)	70 (60–90)	70 (50–93)	A vs. Ctrl *, A vs. C *, A vs. D *

PCOS: Polycystic Ovary Syndrome, BMI: Body Mass Index, WC: Waist Circumference, SBP: Systolic Blood Pressure, DBP: Diastolic Blood Pressure, Ctrl: Controls. **Significance codes:** * *p* < 0.05; ** *p* < 0.01; *** *p* < 0.001. All unlisted comparisons were non-significant (NS) (*p* > 0.05). Results are given as median (minimum–maximum).

**Table 2:** Comparison of metabolic, renal, and urinary parameters between control subjects and PCOS phenotypes (A–D).

	Control Group (*n* = 51)	PCOS Phenotype A (*n* = 96)	PCOS Phenotype B (*n* = 19)	PCOS Phenotype C (*n* = 35)	PCOS Phenotype D (*n* = 30)	PCOS (*n* = 180)	Significant Pairwise Comparisons
Glucose (mg/dL)	87 (73–98)	90 (56–142)	88 (75–109)	88 (60–103)	88 (78–103)	89 (56–142)	All comparisons NS
Insulin (μU/mL)	7.32 (2.80–22.00)	13.70 (3.60–100.50)	13.60 (4.40–35.70)	10.10 (2.50–37.80)	9.60 (2.00–30.50)	12.59 (2.00–100.50)	A > Ctrl ***, B > Ctrl ***, C > Ctrl **, D > Ctrl *; A > C **, A > D **; PCOS > Ctrl ***
HOMA-IR	1.63 (0.58–4.35)	2.96 (0.77–25.57	3.06 (0.92–7.31)	2.30 (0.55–7.54)	2.13 (0.38–6.55)	2.75 (0.38–25.57)	A > Ctrl ***, B > Ctrl ***, D > Ctrl *; A > C **, A > D **; PCOS > Ctrl ***
SCr (mg/dL)	0.65 (0.60–0.90)	0.66 (0.50–1.00)	0.66 (0.60–0.80)	0.66 (0.50–0.80)	0.64 (0.50–0.90)	0.66 (0.50–1.00)	All comparisons NS
CKD-EPI	122.31 (90.12–129.38)	121.79 (78.24–137.38)	119.58 (96.19–132.14)	123.17 (105.39–137.38)	123.42 (82.84–136.42)	122.10 (78.20–137.40)	Ctrl > A *; A < C *
UC (mg/dL)	103.87 (6.50–381.10)	126.31 (15.30–419.30)	120.30 (14.00–330.70)	96.42 (8.10–246.40)	137.82 (6.70–395.00)	122.62 (6.70–419.30)	All comparisons NS
UA (mg/L)	15 (5–468)	22 (5–616)	45 (5–433)	12 (5–253)	15 (4–159)	21 (4–616)	A > Ctrl *, B > Ctrl **; A > C *, B > C **, B > D *; PCOS > Ctrl *
U-ACR mg/g creatinin	12.38 (5.25–426.62)	22.81 (3.97–334.43)	38.95 (5.49–164.72)	17.78 (5.13–172.63)	13.62 (2.52–152.99)	19.66 (2.52–426.62)	B > Ctrl *, B > D *

PCOS: Polycystic Ovary Syndrome, HOMA-IR: Homeostasis Model Assessment of Insulin Resistance, SCr: Serum Creatinine, CKD-EPI: Chronic Kidney Disease Epidemiology Collaboration, UC: Urinary Creatinine, UA: Urinary Albumin, U-ACR: Urinary Albumin-to-Creatinine Ratio, Ctrl: Controls. **Significance codes:** * *p* < 0.05; ** *p* < 0.01; *** *p* < 0.001. All unlisted comparisons were non-significant (NS) (*p* > 0.05). Results are presented as median (minimum–maximum).

**Table 3:** The number and percentage of cases distributed across groups according to categorized urinary albumin-to-creatinine ratio levels.

U-ACR Category	The Control Group (*n* = 51)	Phenotype A (*n* = 96)	Phenotype B (*n* = 19)	Phenotype C (*n* = 35)	Phenotype D (*n* = 30)	Total PCOS (*n* = 180)	p1	p2
<29.9 mg/g creatinine	37 (72.5)	59 (61.5)	7 (36.8)	22 (62.9)	22 (73.3)	110 (61.1)	≥0.05	≥0.05
30–299 mg/g creatinine	12 (23.5)	35 (36.5)	12 (63.2)	13 (37.1)	8 (26.7)	68 (37.8)		
>300 mg/g creatinine	2 (3.9)	2 (2.1)	0 (0)	0 (0)	0 (0)	2 (1.1)		

PCOS: Polycystic Ovary Syndrome, U-ACR: Urinary albumin-to-creatinine ratio, p1: Comparison of subgroups among themselves and with the Control Group, p2: Comparison between patients with or without PCOS. Categorical variables were compared using the Pearson chi-square test, as appropriate. Non-significant values are presented as *p* ≥ 0.05.

**Table 4:** Correlations among age, body mass index, HOMA-IR, and urinary albumin-to-creatinine ratio.

	Age (Year)	BMI (kg/m^2^)	HOMA-IR	U-ACR (mg/g Creatinine)
Age (year)	r = 1	r = 0.235 *p* < 0.001	r = 0.011 *p* ≥ 0.05	r = −0.100 *p* ≥ 0.05
BMI (kg/m^2^)	r = 0.235 *p* < 0.001	r = 1	r = 0.537 *p* < 0.001	r = −0.022 *p* ≥ 0.05
HOMA-IR	r = 0.011 *p* ≥ 0.05	r = 0.537 *p* < 0.001	r = 1	r = −0.007 *p* ≥ 0.05
U-ACR (mg/g creatinine)	r = −0.100 *p* ≥ 0.05	r = −0.022 *p* ≥ 0.05	r = −0.007 *p* ≥ 0.05	

BMI: Body Mass Index, HOMA-IR: Homeostasis model assessment of insulin resistance, U-ACR: Urinary albumin-to-creatinine ratio. Diagonal values represent self-correlations (r = 1). Non-significant values are presented as *p* ≥ 0.05.

## Data Availability

The original contributions presented in this study are included in the article/Supplementary Materials. Further inquiries can be directed to the corresponding author.

## Supplementary Materials

The following supporting information can be downloaded at https://www.mdpi.com/article/10.3390/metabo16070448/s1, Table S1: Hierarchical linear regression predicting log-transformed urinary albumin-to-creatinine ratio (log U-ACR) in the entire study group (*n* = 231).
