# Supplementary material for: Urinary Albumin-to-Creatinine Ratio Across Phenotypes of Polycystic Ovary Syndrome: A Phenotype-Based Evaluation
Source: Metabolites. 2026 Jun 25;16(7):448. doi: 10.3390/metabo16070448 (PMC13413895; doi:10.3390/metabo16070448)
Supplement: Supplementary file 1 [file metabolites-16-00448-s001.zip › metabolites-4225456-supplementary.pdf]

Supplementary Table S1. Hierarchical linear regression predicting log-transformed urinary albumin-to-creatinine ratio (log U-ACR) in the entire study group (n = 231)

Predictor	β Block 1	p Block 1	β Block 2	p Block 2	95% CI Low	95% CI High	VIF
Metabolic Covariates							
**Age**	−0.091	0.184	−0.080	0.246	−0.055	0.014	1.09
**BMI**	−0.024	0.768	−0.058	0.492	−0.035	0.017	1.68
**HOMA-IR**	0.068	0.391	0.044	0.581	−0.046	0.082	1.47
PCOS Phenotypes (Reference: Control Group)							
**Phenotype A**	—	—	0.115	0.224	−0.157	0.666	2.07
**Phenotype B ***	—	—	0.164	**0.029**	0.066	1.240	1.31
**Phenotype C**	—	—	0.019	0.811	−0.415	0.530	1.45
**Phenotype D**	—	—	−0.030	0.703	−0.599	0.405	1.43
Model Fit Statistics							
**R^2**	0.013	—	0.043	—	—	—	—
**ΔR^2**	—	—	0.031	0.134	—	—	—

Abbreviations: VIF: Variance Inflation Factor, CI: Confidence interval, BMI: Body mass index, HOMA-IR: Homeostasis model assessment of insulin resistance.

*Block 1 includes metabolic covariates only; Block 2 adds PCOS phenotype dummy variables (Control = reference).
